# Salinomycin inhibits the tumor growth of glioma stem cells by selectively suppressing glioma-initiating cells

**DOI:** 10.3892/mmr.2014.3027

**Published:** 2014-12-01

**Authors:** TUNAN CHEN, LIANG YI, FEI LI, RONG HU, SHENGLI HU, YI YIN, CHUAN LAN, ZHAO LI, CHUHUA FU, LIU CAO, ZHI CHEN, JISHU XIAN, HUA FENG

**Affiliations:** 1 Department of Neurosurgery, Southwest Hospital, Chongqing 400038, P.R. China; 2 Department of Neurosurgery, Daping Hospital, Chongqing 400042, P.R. China

**Keywords:** glioma, glioma-initiating cells, salinomycin, apoptosis, cell viability

## Abstract

Glioma-initiating cells are a small population of cells that have the ability to undergo self-renewal and initiate tumorigenesis. In the present study, the potential role of salinomycin, a polyether antibiotic, on the suppression of glioma cell growth was investigated. GL261 glioma cells were maintained in a stem-cell-like status [GL261 neurospheres (GL261-NS)] or induced for differentiation [GL261 adherent cells (GL261-AC)]. It was demonstrated that salinomycin significantly reduced the cell viability of GL261-NS and GL261-AC cells in a dose-dependent manner, with a more substantial inhibition of GL261-NS proliferation (P<0.05). The inhibitory effect of salinomycin on cell growth was more effective than that of 1-(4-amino-2-methyl-5-pyrimid l)-methyl-3-(2-chloroethyl)-3-nitrosourea hydrochloride and vincristine (P<0.05). Salinomycin depleted GL261-NS from tumorspheres and induced cell apoptosis. In addition, salinomycin prolonged the median survival time of glioma-bearing mice (P<0.05). Therefore, the present study indicated that salinomycin may preferentially inhibit glioma-initiated cell growth by inducing apoptosis, suggesting that salinomycin may provide a valuable therapeutic strategy for the treatment of malignant glioma.

## Introduction

Malignant glioma is the most common type of primary brain tumor in adults and is associated with a disproportionately high morbidity and mortality (1). Despite advances in understanding the molecular pathogenesis of malignant glioma, the prognosis and therapy of this tumor type remains poor. A subpopulation of glioma cells, termed glioma-initiating cells (GICs), which are brain tumor-initiating cells or glioma stem cells, has been previously identified in glioma (2–4). These cells have the ability to undergo self-renewal and initiate tumorigenesis. They are considered to be responsible for the initiation, propagation and recurrence of tumors. In addition, it appears that tumor stem-like cells may be resistant to numerous conventional cancer therapies, which may explain the limitations of traditional agents in curing human malignancy (4,5). Therefore, therapeutic strategies aimed at targeting these cells may be more effective in providing durable responses.

Salinomycin is a polyether antibiotic isolated from *Streptomyces albus,* which acts as an ionophore with a high affinity for potassium (6,7). In addition to its well-established antimicrobial activities, it has previously been demonstrated to act as a specific inhibitor of breast cancer stem cells (8). Although the mechanism of action of salinomycin remains to be elucidated, it has been reported that salinomycin may serve as a permeability-glycoprotein inhibitor, thus impairing the viability of cancer stem cells (9). By contrast, salinomycin has also been demonstrated to induce apoptosis in cancer cells and overcome apoptotic resistance in human breast cancer cells (10). Nevertheless, the activity of salinomycin in growth suppression and tumorsphere formation in glioma, particularly GICs, remains to be elucidated.

In the present study, the *in vitro* and *in vivo* effects of salinomycin on GL261 glioma cells were investigated. The present study may provide valuable insights into understanding the pathogenesis of GICs and may offer a novel therapeutic approach for the treatment of human malignant glioma.

## Materials and methods

### Reagents

Dulbecco’s modified Eagle’s medium (DMEM)/F12 culture medium was purchased from Life Technologies (Gaithersburg, MD, USA). Fetal bovine serum (FBS) and B27 supplement were purchased from Gibco-BRL (Grand Island, NY, USA). Basic fibroblast growth factor (bFGF) and epidermal growth factor (EGF) were obtained from PeproTech (Rocky Hill, NJ, USA). Normal goat serum was provided by Wuhan Boshide Biotechnology Co., Ltd. (Wuhan, China). Poly-L-lysine, 4′,6-diamidino-2-phenylindole (DAPI), fluorescein isothiocyanate (FITC)-conjugated goat anti-rabbit antibody and Cy3-conjugated goat anti-rabbit antibody were provided by Sigma (St. Louis, MO, USA). Rabbit anti-mouse CD133 monoclonal antibody and glial fibrillary acidic protein (GFAP) polyclonal antibody were purchased from Abcam (Cambridge, MA, USA) and Zhongshan Jinqiao Biotechnology, Ltd., (Beijing, China), respectively. Anti-caspase-3 antibody (rabbit polyclonal against mouse, rat or human) and anti-β-actin antibody were obtained from Abcam Inc. (Cambridge, MA, USA). Cell Counting kit-8 (CCK-8) was purchased from Dojindo Laboratories (Tokyo, Japan).

### Cell culture

GL261 cells were obtained from the American Type Culture Collection (ATCC, Manassas, VA, USA) and were cultured in DMEM/F12 culture medium containing 10% FBS. Cells were maintained at 37°C in a humidified environment containing 5% CO_2_. The medium was changed every 4 days. To induce the formation of neurospheres (NS), FBS was gradually withdrawn from culture medium in a gradient reduction pattern (10, 5, 2 and 0%). Cells were then maintained in serum free DMEM/F12 medium supplemented with 20 ng/ml bFGF, 20 ng/ml EGF, B27 (1X), 2 mM L-glutamine and 4 U/l insulin. The floating cells formed NS-like clones and secondary spheres derived from single cells of these clones were used to induce cell differentiation. To induce differentiation, the GL261-NS cells were seeded onto coverslips and cultured in DMEM/F12 culture medium containing 10% FBS. These cells were defined as GL261 adherent cells (AC).

### Immunocytochemistry analysis

The spheres were placed onto coverslips precoated with poly-L-lysine (Sigma) and then fixed with 4% paraformaldehyde for 20 min at room temperature. Cell samples were blocked with normal goat serum and then incubated with the following respective primary antibodies: Rabbit anti-mouse CD133/1 monoclonal antibody (1:50 dilution) and GFAP polyclonal antibody (1:100 dilution) overnight. The cells were then washed three times in phosphate-buffered saline (PBS) and then stained with Cy3-conjugated goat anti-rabbit (1:100 dilution) or FITC-conjugated goat anti-rabbit secondary antibody (1:50 dilution) for 30 min. Cell samples were then counterstained with 100 mg/ml DAPI for 10 min to visualize nuclei and then analyzed with a confocal laser scanning microscope (Leica, Mannheim, Germany).

### Determination of cell viability

The effect of drug treatment on cell viability was determined using the CCK-8 kit. Briefly, cells were seeded onto a 96-well-plate and then treated 24 h after seeding with different concentrations (0.01, 0.03, 0.1, 0.3, 1, 3, 10, 30 μM) of drug. Dimethyl sulfoxide (DMSO) was used as a negative control. Four days after drug incubation, 10 μl of thawed CCK-8 solution was added to each well. Plates were incubated for 4 h at 37°C and the absorbance was read at 450 nm with a reference wavelength of 600 nm using the Thermo Scientific™ Varioskan™ Flash Multimode Reader (Thermo Fisher Scientific, Waltham, MA, USA).

### Colony formation assay

In order to evaluate the effects of agents on the colony formation of GL261-NS, cells were seeded onto a 96-well plate at a density of 1×10^3^ cells/ml. The cells were then incubated with 0.1 μM salinomycin, 30 μM 1-(4-amino-2-methyl-5-pyrimidyl)-methyl-3-(2-chloro ethyl)-3-nitrosourea hydrochloride (ACNU) or 10 μM vincristine (VCR) dissolved in DMSO. DMSO was used as a negative control. A total of 12 wells were assessed for each treatment group. Six days after incubation, the colony forming ability of cells were examined and the number of colonies was counted under an Olympus CX22 microscope (Olympus Corp., Inc., Tokyo, Japan).

### Flow cytometric analysis

Apoptotic cell death was measured using the Annexin V-FITC/propidium iodide Apoptosis Detection kit (Dojindo Molecular Technologies, Inc., Kunamoto, Japan) according to the manufacturer’s instructions and assessed on a fluorescence activated cell sorting Calibur instrument (Beckman Coulter Inc., Miami, FL, USA). Data were analyzed using CellQuest software (Becton-Dickinson, San Jose, CA, USA).

### Western blot analysis

Cells were washed with ice-cold PBS and then lysed in ice-cold lysis buffer (50 mM Tris, pH 7.4, 150 mM NaCl, 1% Triton X-100, 1 mM EDTA, 1 mM ethylene glycol tetraacetic acid, 1 mM phenylmethylsulfonyl fluoride, 10 μg/ml aprotinin, 10 μg/ml leupeptin, 1 mM sodium orthovandate and 1 mM NaF) for 30 min. Following this, samples were centrifuged at 16,000 × g at 4°C for 30 min. The supernatant was collected and the protein concentration was determined using the Bradford protein assay. Total cell protein was separated by 12% SDS-PAGE and transferred onto a polyvinylidene difluoride membrane (Millipore, Billerica, MA, USA). Membranes were blocked with 5% non-fat milk then incubated with anti-caspase-3 primary antibody at 4°C overnight. On the following day, the membrane was washed with 0.1% Tween 20 in PBS and probed with horseradish peroxidase-conjugated secondary antibody for 1 h at room temperature. The bound antibody complexes were detected using an electrochemiluminesence reagent (GE Healthcare, Amersham, UK). β-actin was used as an internal control. Band images were analyzed with a gel imaging analysis system (Kodak ID, Kodak, Rochester, NY, USA). The intensities of the immunoreactive bands were quantified by densitometric analysis.

### Establishment of a tumor-bearing animal model

A total of 40 six-week-old specific pathogen free level C57BL/6 male mice weighing 22±2 g were obtained from the Laboratory Animal Center, Third Military Medical University (Chongqing, China). Animals were randomly divided into the following four groups each including 10 animals: Sham-surgery, sham-surgery plus salinomycin, tumor-bearing plus placebo and tumor-bearing plus salinomycin. In tumor-bearing groups, animals received orthotopic transplantation of GL261-NS cells. Briefly, mice were anesthetized with 400 mg/kg chloral hydrate via intraperitoneal injection. Animal experiment procedures were approved by the Ethics Committee of Southwest Hospital, Third Military Medical University (Chongqing, China). The cell density was adjusted to 2×10^6^ cells/ml in serum free DMEM/F12 culture medium and then 5 μl of cell suspension was injected into the right caudate nucleus. Mice were placed on a stereotaxic instrument. Following sterilization and skin incision, a hole with a diameter of 3 mm was made with a micro-electrical drill (RWD Life Science, Inc., Shenzhen, China) on the skull at the position of 1.4 mm anterior to the anterior fontanel and 2.0 mm lateral to the sagittal suture. Stereotaxic coordinates were obtained from the bregma and the dura mater according to a mouse brain atlas (11). In the sham surgery group, mice received 1 ml of normal saline by intraperitoneal injection 24 h after cell injection and for salinomycin treatment, mice were subjected to intraperitoneal administration of salinomycin. The two groups of animals received daily injections for 10 days. The animal survival time was assessed for up to 60 days post-surgery. Animal experiments were conducted according to the guidelines of the Institutional Committee for Laboratory Animal Usage. The study was approved by the Laboratory Animal Welfare and Ethics Committee of the Third Military Medical University (Chongqing, China).

### Statistical analysis

Statistical analyses were performed using the SPSS 13.0 statistical software package (SPSS, Inc., Chicago, IL, USA). Data are presented as the mean ± standard error of the mean. The results were analyzed using a one-way analysis of variance with Fisher’s Least Significant Difference test. LogRank (Mantel-Cox) analysis was used to compare the significance of median survival time of animals. P<0.05 was considered to indicate a statistically significant difference.

## Results

### Identification and characterization of cultured GL261-NS and GL261-AC cells

**Figure 1:**
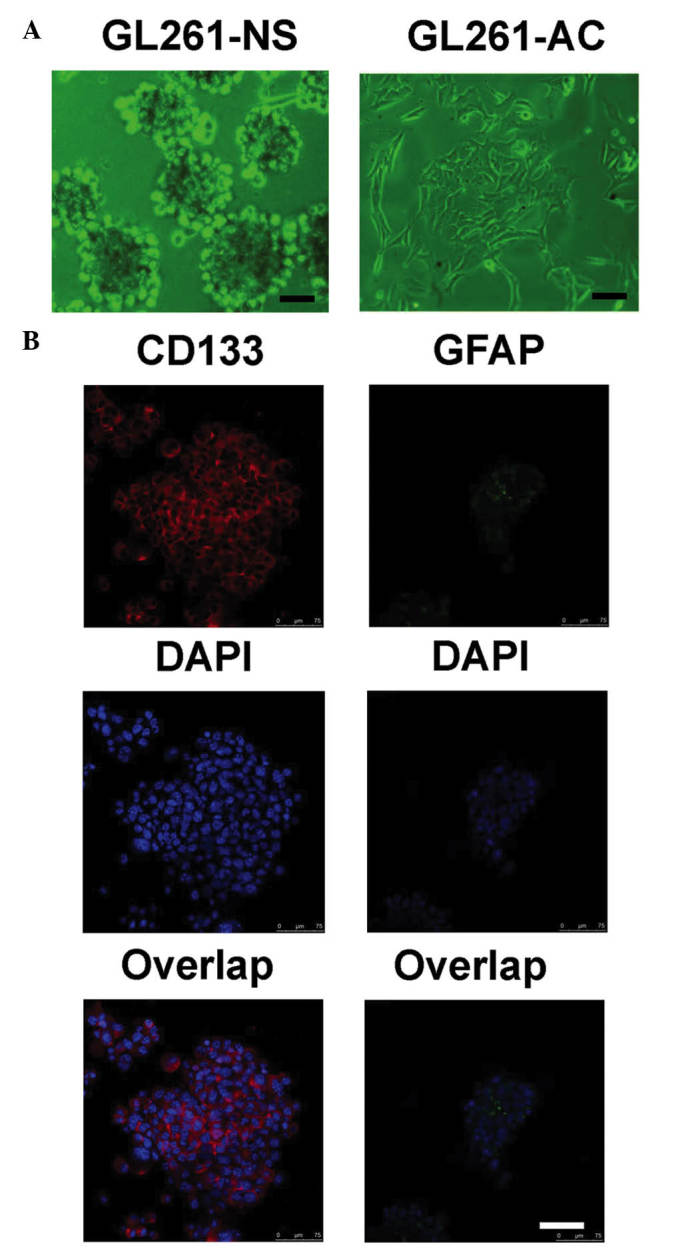
GL261-NS and GL261-AC cells cultured *in vitro*. (A) Cells were visualized under phase contrast microscopy. Scale bar=50 μm. (B) Immunofluorescence of stem cell maker CD133 (red) and differentiated glia cell marker, GFAP (green). Cell nuclei were counterstained with DAPI (blue). Scale bar=75 μm. GFAP, glial fibrillary acidic protein; NS, neurospheres; AC, adherent cells; DAPI, 4′,6-diamidino-2-phenylindole.

Neurosphere-like clones formed when the cells were cultured in serum-free culture medium supplemented with bFGF, EGF, L-glutamine and insulin (Fig. 1A). In addition, abundant expression of the stem cell marker, CD133, was observed in these neurosphere-like GL261-NS cells (Fig. 1B). In addition, the level of GFAP expression, which is a biomarker for differentiated glial cells, was relatively low. In order to induce cell differentiation, the culture medium was changed and cells were maintained in DMEM/F12 culture medium containing 10% FBS. The majority of the cells became adherent and began to differentiate, which were defined as GL261-AC cells.

### Salinomycin reduces the cell viability of GL261-NS and GL261-AC cells

**Figure 2:**
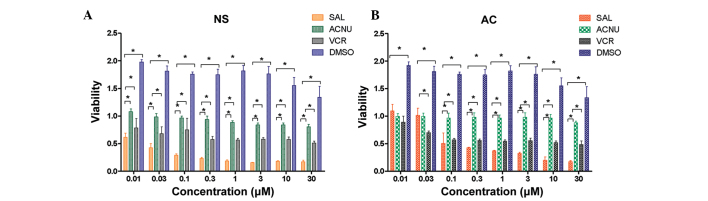
Salinomycin reduces the viability of GL261-NS and GL261-AC cells. (A) GL261-NS and (B) GL261-AC cells cultured in a 96-well plate were treated with different concentrations of SAL, ACNU or VCR for 96 h. DMSO was used as a negative control. Cell viability was determined using the CCK-8 assay. Data were quantified from 12 wells. ^*^P<0.05. Sal, salinomycin; ACNU, 1-(4-amino-2-methyl-5-pyrimidyl)-methyl-3-(2-chloroethyl)-3-nitrosourea hydrochloride; VCR, vincristine; DMSO, dimethyl sulfoxide; NS, neurospheres; AC, adherent cells.

The effects of salinomycin on the cell viability of GL261-NS and GL261-AC cells were then determined. Two commonly used anti-cancer agents, ACNU and VCR, were also used as controls. As shown in Fig. 2, salinomycin (0.01 μM-30 mM) significantly decreased the cell viability of GL261-NS and GL261-AC cells in a dose-dependent manner (P<0.05, compared with the DMSO control). Salinomycin appeared to preferentially inhibit the cell growth and viability of GL261-NS cells compared with GL261-AC cells with IC50 values of 0.06 and 0.58 μM, respectively. In addition, a salinomycin concentration of 0.1 μM or higher was more effective in decreasing cell viability than ACNU or VCR (P<0.05).

### Salinomycin suppresses GL261-NS colony formation

**Figure 3:**
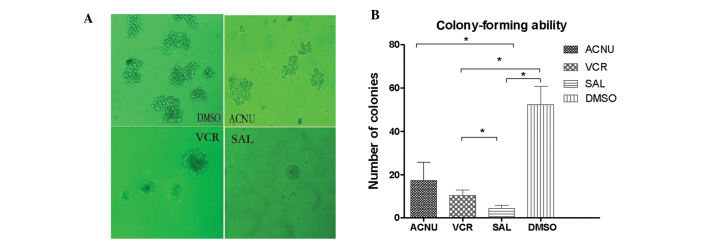
Salinomycin suppresses the colony formation of GL261-NS. Cells were cultured in 0.1 μM SAL, 30 μM ACNU or 10 μM VCR for 6 days. (A) Morphology of colonies following drug treatment. (B) Number of colonies in each well of a 96-well plate was counted. Data were quantified from 12 wells. ^*^P<0.05. Sal, salinomycin; ACNU, 1-(4-amino-2-methyl-5-pyrimidyl)-methyl-3-(2-chloroethyl)-3-nitrosourea hydrochloride; VCR, vincristine; DMSO, dimethyl sulfoxide.

The effect of drug treatment on GL261-NS colony formation was then assessed. Although 30 μM ACNU or 10 μM VCR significantly decreased the sphere-forming ability of GL261-NS cells after 6 days of treatment, the administration of only 0.1 μM salinomycin markedly inhibited GL261-NS colony formation (SAL, 4.31 ± 1.53%; ACNU, 17.31 ± 8.53%; VCR, 10.41 ± 2.53%; DMSO, 52.31 ± 8.53%; P<0.05, SAL compared with the other three groups; Fig. 3). These results indicate that salinomycin is significantly more effective at reducing colony formation than standard chemotherapy.

### Salinomycin promotes GL261-NS apoptosis

**Figure 4:**
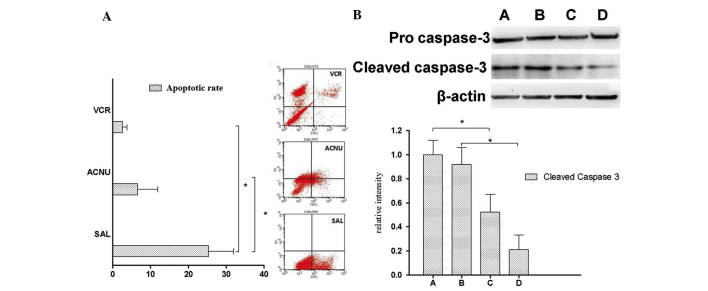
Salinomycin promotes the apoptosis of GL261-neurosphere cells. (A) Cell apoptosis was evaluated by flow cytometry using Annexin V-fluorescein isothiocyanate/propidium iodide double staining. Data were quantified from three independent experiments. ^*^P<0.05. (B) Cells were treated with (A and C) 0.1 or (B and D) 10 μM SAL (A and B) for 12 or (C and D) 24 h. Protein expression of pro-caspase-3 and cleaved caspase-3 (activated caspase-3) was examined by western blot analysis. β-actin was used as an internal control. Data were quantified from three independent experiments. ^*^P<0.05. Sal, salinomycin; ACNU, 1-(4-amino-2-methyl-5-pyrimidyl)-methyl-3-(2-chloroethyl)-3-nitrosourea hydrochloride; VCR, vincristine.

Following this, the effect of salinomycin on GL261-NS apoptosis was assessed. The administration of SAL markedly increased apoptotic cell death (P<0.05; Fig. 4A). The expression of the apoptotic cascade protein caspase-3 was further examined in GL261-NS cells following 12 or 24 h of salinomycin treatment. Significantly elevated expression of activated caspase-3 was observed in cells treated with 10 μM compared with 0.1 μM salinomycin (P<0.05; Fig. 4B). The level of activated caspase-3 was also significantly reduced after 24 h of treatment compared with 12 h (P<0.05). Collectively, these data indicate that salinomycin promotes apoptosis of GL261-NS cells.

### Salinomycin prolongs the median survival time of tumor-bearing mice

**Figure 5:**
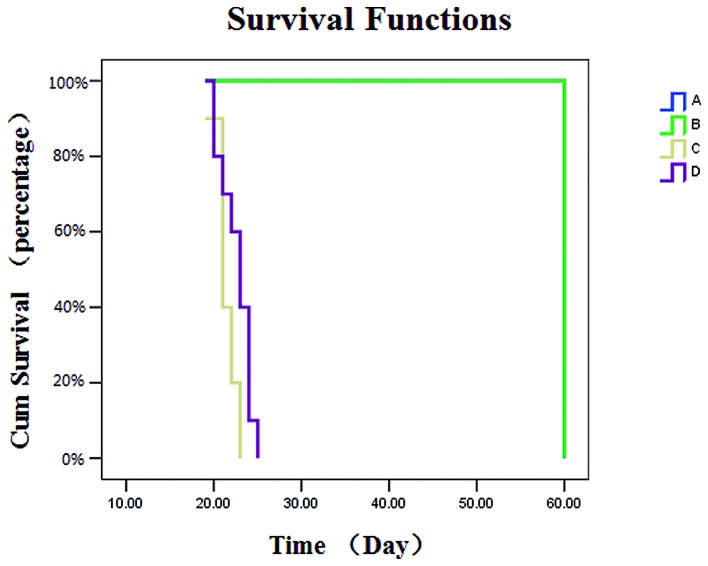
Survival curves of glioma xenograft animals treated with salinomycin. Animals were divided into four groups of 10 animals each: (A) Sham-surgery, (B) sham-surgery plus salinomycin, (C) tumor-bearing plus placebo and (D) tumor-bearing plus salinomycin. The survival time of animals was recorded and plotted.

In order to determine the *in vivo* effects of salinomycin on the survival time of tumor-bearing mice, xenografts were treated with salinomycin and the overall survival rate was monitored. Treatment with salinomycin significantly prolonged the median overall survival of mice injected with GL261-NS cells compared with untreated controls (23 vs*.* 21 days, respectively; P<0.05; Fig. 5). All animals in the sham-surgery and sham-surgery plus salinomycin groups survived for the entire study period (60 days). These findings suggest that salinomycin may be an effective therapy for targeting GICs in glioma, reducing tumor burden and extending survival.

## Discussion

GICs are a small population of cells that have the ability to undergo self-renewal and recapitulate the original tumor *in vivo*. Accumulating evidence indicates that GICs express the cell surface protein CD133 (prominin-1) (12–16). However, certain CD133-negative cells also possess tumor-initiating potential (17,18), indicating the complexity in the identification of cancer cells and underscoring the requirement for more refined biomarkers. In the present study, morphological identification accompanied with biomarker examination was applied to identify GICs from the GL261 cell line. The results demonstrated that in stem-cell culture medium, GL261 glioma cells formed NS-like clones and expressed a high level of CD133. However, these cells could be induced to differentiate in culture medium supplemented with FBS and the majority of the differentiated GL261-AC cells became adherent and expressed the glial cell marker GFAP. These data confirmed the characteristics of the cultured GL261-NS cells (GICs) and GL261-AC cells (differentiated cells) and our ability to induce these effects *in vitro*. In addition, GL261-NS was significantly more aggressive compared with GL261-AC *in vivo* and this effect was enhanced further in syngeneic mice compared with immunodeficient mice as previously reported (19).

Alkylating agents, including temozolomide and nitrosourea derivatives, for example ACNU, remain the most commonly used chemotherapy agents for the treatment of patients with malignant glioma. However, intrinsic or acquired chemoresistance to alkylating agents is recognized as the major cause of treatment failure (20). In addition, plant-derived anticancer compounds, including vincristine and vinblastine, are antimitotic drugs that are also commonly used in anti-cancer therapy. Nevertheless, these agents have limited utility due to the development of drug resistance. Tumor stem cells have been reported to be intrinsically resistant to conventional chemotherapies. These cells can regenerate from the original tumor eradicated by such treatments, ultimately leading to tumor recurrence (21). Therefore, the development of novel treatment approaches targeting this population of cells appears to be critical for more effective chemotherapies.

Salinomycin has been verified to be a specific inhibitor of breast cancer stem cells (8), however, its anti-cancer activity against malignant glioma and GICs remains to be elucidated. The current results demonstrated that salinomycin significantly reduced the cell viability of GL261-NS and GL261-AC in a dose-dependent manner, with a more substantial inhibition of GL261-NS proliferation, indicating that salinomycin may efficiently target GICs *in vitro*. In addition, salinomycin eventually induced cell death by promoting apoptosis of GL261-NS cells. Fuchs *et al* previously reported that salinomycin induced apoptosis and overcame apoptotic resistance in human cancer cells (10). Notably, salinomycin activated a distinct apoptotic pathway not associated with cell cycle arrest, the tumor suppressor protein p53 or caspase activation, which may contribute to the apoptotic resistance observed in cancer cells (10). By contrast, Kim *et al* identified that salinomycin induced apoptosis of human prostate cancer cells by elevating the intracellular reactive oxygen species (ROS) level, decreasing mitochondrial membrane potential, triggering cytochrome c release to the cytoplasm and activating caspase-3 (22). Consistent with this study, it was identified that salinomycin induced cell apoptosis of GL261-NS cells by inducing caspase-3 activity. Future studies are to continue to examine the exact mechanism involved in salinomycin-mediated GICs apoptosis.

In conclusion, the present study demonstrated that salinomycin significantly reduces cell viability of GL261-NS and GL261-AC cells, with a more substantial inhibition of GL261-NS proliferation. It depletes GL261-NS from tumorspheres and induces apoptotic cell death. Additionally, salinomycin prolongs the median survival time of glioma-bearing mice compared with control animals, suggesting that the drug may represent a valuable cancer therapeutic option for the treatment of malignant glioma.
